# The roles of extracellular vesicles in the development, microenvironment, anticancer drug resistance, and therapy of head and neck squamous cell carcinoma

**DOI:** 10.1186/s13046-021-01840-x

**Published:** 2021-01-21

**Authors:** Xueying Wang, Junnan Guo, Pingyang Yu, Lunhua Guo, Xionghui Mao, Junrong Wang, Susheng Miao, Ji Sun

**Affiliations:** 1 Department of Head and Neck Tumors, Harbin Medical University Cancer Hospital, No. 150, Haping Road, Nangang District, 150000 Harbin, Heilongjiang, People’s Republic of China; 2 The First Department of Colorectal Surgery, Harbin Medical University Cancer Hospital, No. 150, Haping Road, Nangang District, 150000 Harbin, Heilongjiang, People’s Republic of China

**Keywords:** Head and neck squamous cell carcinoma, Extracellular vesicles, EXOs, Tumour microenvironment, Drug resistance

## Abstract

Head and neck squamous cell carcinoma (HNSCC) is one of the main malignant tumours affecting human health, mainly due to delayed diagnosis and high invasiveness. Extracellular vehicles (EVs) are membranous vesicles released by cells into the extracellular matrix that carry important signalling molecules and stably and widely exist in various body fluids, such as plasma, saliva, cerebrospinal fluid, breast milk, urine, semen, lymphatic fluid, synovial fluid, amniotic fluid, and sputum. EVs transport almost all types of bioactive molecules (DNA, mRNAs, microRNAs (miRNAs), proteins, metabolites, and even pharmacological compounds). These “cargoes” can act on recipient cells, reshaping the surrounding microenvironment and altering distant targets, ultimately affecting their biological behaviour. The extensive exploration of EVs has deepened our comprehensive understanding of HNSCC biology. In this review, we not only summarized the effect of HNSCC-derived EVs on the tumour microenvironment but also described the role of microenvironment-derived EVs in HNSCC and discussed how the “mutual dialogue” between the tumour and microenvironment mediates the growth, metastasis, angiogenesis, immune escape, and drug resistance of tumours. Finally, the clinical application of EVS in HNSCC was assessed.

## Background

HNSCC is the sixth most common cancer worldwide [1]. Approximately 10 % of HNSCC patients are initially diagnosed with metastatic disease, and approximately half of them will relapse even if treated early [2, 3]. The head and neck region includes the oral cavity, larynx, and pharynx, and all structures are covered with squamous epithelium. Therefore, up to 90 % of head and neck tumours are squamous cell carcinomas [4]. Tobacco use, alcohol consumption, human papillomavirus (HPV) infection and some genetic alterations are risk factors in the development of HNSCC [5–7]. Despite many innovations in HNSCC treatment strategies and molecular targeted drugs, the overall 5-year survival rate is still only approximately 60 % [8, 9]. Therefore, the molecular mechanism of tumorigenesis and the screening of accurate biological markers are major challenges and opportunities for further elucidation of HNSCC.

The tumour microenvironment is composed of stromal cells, endothelial cells, immune cells and other complex components. EVs and EXOs (EXOs) are well known for their cell-cell communication during tumour development. With the analysis of EVs cargo, the function of EVs in tumours has been gradually revealed, and their application in the early diagnosis and treatment of cancer is being explored. Although the veil of EVs has not been fully lifted, with continuous exploration in this field, we believe that EVs will be applied in clinical practice in the immediate future. In this review, we summarize and update the pivotal role of tumour-derived EVs (TDE) in regulating HNSCC development, metastasis, immune escape, and drug resistance. We also describe the multifaceted functions of tumour microenvironmental-derived EVs in HNSCC. In addition, the potential applications of EXOs as non-invasive biomarkers in the early diagnosis and treatment of HNSCC are discussed.

### Biogenesis and classification of EVs

**Fig. 1:**
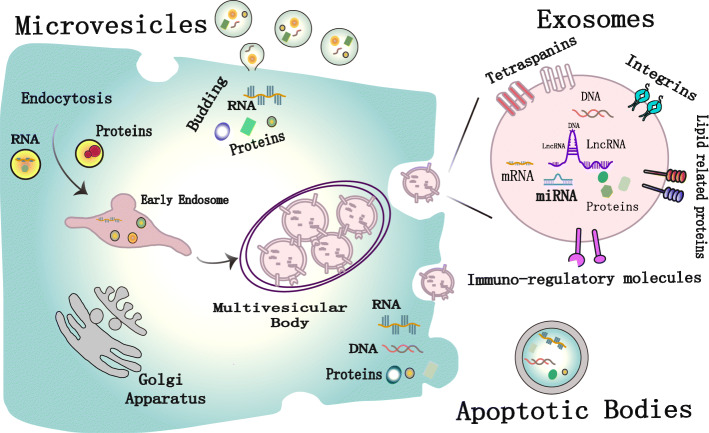
Biogenesis of extracellular vesicle (EV) subtypes, termed exosomes, microvesicles and apoptotic bodies. Exosomes are intraluminal vesicles which are released when a multivesicular body fuses with the cell membrane through exocytosis. Microvesicles are formed by outward shedding of the cell membrane into extracellular space. Apoptotic bodies are generated when cells undergo apoptosis

EVs are produced by many types of cells, such as tumour cells, immune cells and epithelial cells, and are released into the tumour microenvironment(TME) [10]. According to their cell compartment origin, diameter and surface protein markers, they can be divided into three subgroups (Fig. 1): EXOs (40–100 nm), micro vesicles(MVs) (50-1000 nm) and apoptotic bodies (ABs) (50-2000 nm) [11], and the first two are often combined for research [12].

EXOs are small EVs subtypes related to the pathway of endosome biogenesis [13]. The formation of EXOs begins when the early endosomal membranes sprout inward to form intraluminal vesicles [14]. The ubiquitin binding region of endosomal sorting complex required for transport-0 (ESCRT-0) first recognizes and isolates the ubiquitin protein on the endosomal membrane. ESCRT-I and ESCRT-II are then recruited to interact with ESCRT-0 and promote the inward budding of the chelating complex formation. Then, ESCRT-III and other related proteins (such as vacuolar protein sorting 4 (VPS4) and VPS20-associated 1) mediate the division of the inner bud and release the vesicles into the intracellular body cavity [15]. Stuers et al. reported that the depletion of ESCRT-0, -I, -II and -III did not completely block the formation of vesicles in the lumen, indicating that there is a pathway that does not rely on ESCRT [16]. As the early endosomes mature to the late endosomes[17], endosomes with intraluminal vesicles are called multivesicular bodies (MVBs). Most MVBs move toward the plasma membrane, then fuse with the plasma membrane, and then release Intraluminal vesicles, that is, EXOs enter the extracellular space, and the remaining MVBs will fuse with lysosomes, exposing the luminal vesicles to hydrolytic enzymes for catabolism and further degradation [18]. Recent studies have shown that the maturation and differentiation of endosomes are regulated by many pathways of ubiquitin ligase ring finger protein 26 and Rab guanosine triphosphatase [19, 20].

The size of MVs is usually larger than that of EXOs, and MVs flow directly from the plasma membrane through endocytosis [21]. Compared with the biogenesis of EXOs, there are fewer studies on MVs. Plasma membrane bending is an important step in the formation of MVs [22]. Nabhan and colleagues proposed that protein 1 containing the arrestin domain is essential for the formation and release of microcapsules by transporting tumour susceptibility gene 101 from the endosome to the plasma membrane and causing the plasma membrane to bend [23]. Another model by Stachowiak and colleagues believes that protein-protein crowding is important for bending the plasma membrane, rather than protein-specific pathways, because they observed that at higher concentrations, even green fluorescent protein (GFP) that is not related to membrane curvature. GFP can also cause membrane bending [24]. The level of extracellular Ca ^2+^ also significantly affects the production of MVs [25]. One possible reason is the action of Ca^2+^-dependent enzymes, such as amino phospholipid translocases, which cause an asymmetric rearrangement of plasma membrane phospholipids, which is conducive to the curvature of the plasma membrane of MVs buds [26, 27]. This aspect has been used to promote the production of MVs. Researchers treat red blood cells with calcium ionophores to promote the production of EVs for therapeutic purposes [28].

Abs are usually larger than MVs, and will occur when blebbing through the plasma membrane during programmed cell death [29, 30]. During the execution stage of apoptosis, cells undergo a large number of morphological changes, including cell contraction and cytoskeleton rupture, which leads to blistering of ABs [31, 32]. Contrary to the traditional view that ABs are just random fragments of dead cells, ABs are increasingly regarded as important immunomodulators and disease biomarkers [33, 34].

In the TME, EVs can be released from the cell membrane in various cell types, and their contents include various DNA molecules, mRNAs, microRNAs (miRNAs), long noncoding RNAs (lncRNAs), circRNAs, proteins, metabolites, and even pharmacological compounds. The products are even pharmacological compounds. These biologically active substances are important carriers of cell communication and play a vital role in the progression of HNSCC.

### Roles of tumour‐derived EVs in HNSCC

#### Profile of TDE in HNSCC

EVs are a subcellular structure of vesicles enclosed by a phospholipid bilayer. Numerous studies have shown that the cells of virtually all organisms (from prokaryotes to eukaryotes) can release EVs to the extracellular environment in an autocrine or paracrine manner [12]. Currently, EVs have gone from being considered garbage dumpsters to being important carriers of cellular signals.

In cancer patients, EVs are located in body fluids and the TME. They can directly interact with autologous cancer cells within 2 h and then be internalized by them at 24 h as messengers transfer between HNSCC cells to enhance tumour growth [35, 36]. As mentioned earlier, EVs include different subgroups. Distinguishing EV subpopulations is important since intracellular mechanisms leading to MVs and EXOs production are distinct, and each EVs subtype presents a specific protein signature [37, 38], suggesting different effects on target cells. Proteomic analyses have revealed that EV-derived proteins of tumour necrosis factor (TNF) receptor associated protein 1(TRAP1), epidermal growth factor receptor (EGFR), heat shock protein 90 (HSP-90), desmonglein-2 (Dsg-2) and matrix metalloprotein-2/9/13 (MMP) mRNA were significantly overexpressed in HNSCC [39–42]. Noncoding RNAs in HNSCC-derived EVs are involved in the regulation of tumour progression [43, 44]. Wang et al. demonstrated that the expression of exosomal miRNANA-21 and HOX transcript antisense RNA (HOTAIR) was markedly higher in patients with HNSCC than in those with non-malignant tumours and significantly correlated with clinical parameters of HNSCC [45]. Furthermore, recent studies have shown that miRNA-21-enriched EXOs increase the expression of the Snail and Vimentin proteins and downregulate E-cadherin levels in tumour cells, suggesting that oral squamous cell carcinoma (OSCC) can create a niche for distant transfer [45, 46]. After a comparison of the EXOs secreted by both HNSCC and their normal cells, the miRNA expression profiles of HPV-associated HNSCC were identified by Sonja et al. miRNA libraries showed that the highly expressed miRNAs were different among EXOs from HPV + and HPV- associated tumour cells, and 8 miRNAs that were overexpressed in HPV (+) EXOs and 14 that were overexpressed in HPV (-) EXOs were identified. The analysis of miRNAs in HPV (+) vs. HPV (-) EXOs is currently in progress [47]. All these findings suggest that EVs have their own characteristics and functions and should be considered potential anticancer therapeutic targets.

#### TDE affect tumour growth

**Fig. 2:**
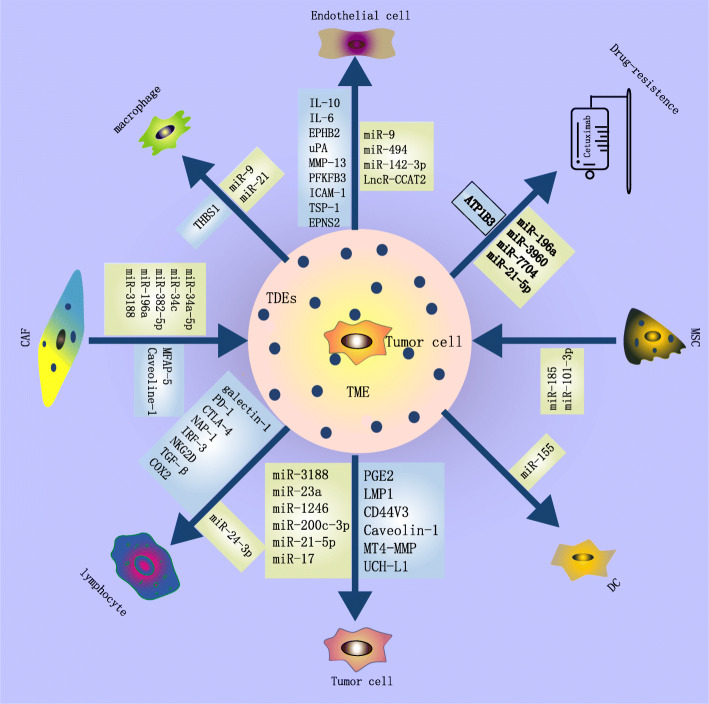
A schematic representation of EVs from different sources in the regulation of tumor cells, a variety of stromal cells and drug resistence

Several proteins and miRNAs that are contained in TDE promote HNSCC growth (Fig. 2). Li et al. demonstrated that exosomal miRNA-3188 can influence the proliferation of HNSCC cells by directly targeting B-cell lymphoma 2 (BCL2) in vitro and in vivo [48]. Myeloid-derived suppressor cells (MDSCs) promote tumour growth, and in vivo MDSC mediated promotion of tumour progression is dependent on tumour EXOs prostaglandin E2 (PGE2) and transforming growth factor-β (TGF-β) molecules. Further experiments show that antibodies against exosomal PGE2 and TGF-β block the activity of these EXOs on MDSCs induction and therefore attenuating MDSCs mediated tumour-promoting ability. This could be useful for the development of specific targeted tumour treatment strategies [49]. EVs derived from HNSCC cells can stimulate the proliferation of tumour cells by delivering exosomal 6-phosphofructo-2-kinase/fructose-2,6-biphosphatase (PFKFB3), Sonic Hh (Shh) and other angiogenic proteins and activating the relevant model pathway to induce endothelial proliferation and tube formation [50, 51].Similarly, nasopharyngeal carcinoma (NPC) cell-derived exosomal miRNA-23a directly targets the targeting testis-specific gene antigen (TSGA10) region to accelerate endothelial cell generation and migration to regulate tumour growth [52]. Shinya Sento et al. proved that HNSCC-derived EXOs can self-absorb or be absorbed by surrounding tumour cells and then promote cell proliferation and invasion by activating the protein kinase B (AKT), mitogen-activated protein kinase(MAPK) / extracellular signal-regulated kinase (ERK), and c-Jun N-terminal kinases (JNK) signalling pathways [53]. However, in the presence of heparin, the uptake of EXOs by OSCC cells and subsequent tumour progression was abrogated. These data suggest that OSCC cell-derived EXOs might be a novel therapeutic target and the use of heparin to inhibit the uptake of OSCC-derived EXOs by OSCC cells may be useful for treatment [53]. Notably, highly metastatic and invasive OSCC can transport EXOs-derived miRNA-1246 and miRNA-200c-3p to parental OSCC cells, which could target and bind the DENN/MADD Domain Containing 2D (DENND2D) and chromodomain helicase DNA 9/Werner, thereby promoting tumour cell proliferation, metastasis and invasion [54, 55]; therefore, it is important to understand the molecular mechanisms of invasion and subsequent metastasis not only to prevent cancer progression but also to detect new therapeutic targets in OSCC.

Viral infection plays an increasingly important role in HNSCC. Several studies have confirmed that HPV (+) cancer cells can identify the expression of tumour-related genes and proteins in EVs to exert proliferative, antiapoptotic and ant senescent effects on surrounding cells [56, 57]. Moreover, continuous expression of E6/E7 protein in HPV (+) cancer cells can alter the miRNA pool intracellularly and in EXOs; For example, the upregulated miRNA-17 family can inhibit P53/P21 expression levels and regulate tumour cell proliferation[58]. Epstein-Barr virus (EBV) can hijack TDE production to regulate cell-to-cell communication and package viral components, such as latent membrane protein 1(LMP1) and cluster of differentiation 63 (CD63), which regulate the TME and promote tumour development [59, 60]. Mechanistically, EVs released by EBV-positive NPC activate the ERK and phosphatidylinositol 3 kinase (PI3K) / AKT proliferation pathways in cancer and endothelial cells via selective transport of LMP1, EGFR, and virus-encoded miRNAs [61, 62]. This provides a basis to explore their potential as a source of novel tumour biomarkers and their possible role in communications between malignant and non-malignant cells. In general,these findings are of great important for future studies on the physiological and pathological mechanisms of extracellular vesicle biogenesis, protein transport and signal transduction, especially for virus-associated tumorigenesis.

#### TDE promote metastasis

The most important prognostic determinant of HNSCC tumours is considered the presence of lymph node metastases, since lymphatic metastatic spread correlates with a significant decrease in the survival rate of patients [63]. TDE and communication with the TME are critical factors in tumour metastasis [64]. Theodoraki et al. demonstrated that CD44v3 protein-carrying TDE are correlated with higher disease stages and lymph node metastasis; thus, TDE could potentially serve as a non-invasive biomarker of cancer progression [65]. Studies have shown that ubiquitin C-terminal hydrolase L1 (UCH-L1) promotes motility of metastatic HNSCC as well as of extracellular vesicle-mediated transfer of the viral invasive factor LMP1 [66]. Further, Kobayashi et al. proved that soluble inhibitors of UCH-L1 are effective in reducing lymph node metastasis of HNSCC; therefore, soluble inhibitors of UCH-L1 offer potential forms of treatment for invasive carcinomas, including EBV-positive malignancies [67].

Noncoding RNAs and hypoxic-derived EXOs are involved in the regulation of tumour progression [68, 69].High expression of lysyl oxidase like 2 (LOXL2) was previously found in metastatic human HNSCC cells in a mouse model of lymph node metastasis. Immunoblot analyses revealed that LOXL2 was present in the serum exosomal fractions from three HNSCC patients, and Sanada et al. observed approximately threefold higher levels of LOXL2 in the HNSCC patients compared with three healthy volunteers. The hypoxic microenvironment may stimulate tumour cells to generate miRNA-21-rich EXOs that are delivered to normal cells to promote premetastatic behaviours [70]. Similarly, bioinformatics analysis has shown that miR-21-enriched EVs are associated with increased HNSCC metastasis and poor survival [71]. On the other hand, precancerous cells usually exhibit epithelial-mesenchymal transition (EMT), promoting migration and invasion of the cells within the tumour milieu [72]. It is currently understood that there are three main ways to mediate the occurrence of EMT. First: miRNA-21-enriched EXOs may create a niche for distant transfer via the EMT [46]. Second: tumour-derived EVs upregulate N-cadherin, downregulate glioma-associated oncogene homolog 1 (GLI-1), and promote EMT, and eventually accelerating cell metastasis. Third: epidermal growth factor (EGF) stimulates the secretion of EGFR-EVs by OSCC cells and therefore may increase the downstream RAS-MEK-ERK signalling pathway and promote EGF-driven EMT progression [73, 74]. Hypoxia causes HNSCC cells to secrete caveolin-1, through trafficking by EVs, which is a direct transcriptional target of hypoxia-inducible factor-1α (HIF-1α) and HIF-2α. EVs carrying caveolin-1 can construct a pseudo hypoxic environment and contribute to tumour growth and migration [40, 75]. A study by Huang et al. reported that SLUG regulated the expression of MT4-MMP under hypoxia, which promoted the invasiveness of HNSCC cell lines [76].

### Roles of tumour‐derived EVs in the HNSCC microenvironment

In this section, we will focus on the impact of EVs on the TME. As a medium, EVs play a crucial role in the communication between tumour cells and the TME (Fig. 2). The TME contains complex components, such as extracellular matrix, immune cells, stromal cells, endothelial cells, blood vessels, and nonepithelial cells, such as fibroblasts. TDE play a critical role in the establishment of the TME.

#### The effects of TDE on angiogenesis

As early as 2015, Chan et al. reported that EXOs derived from NPC C666-1 cells could markedly enhance the tubulogenesis, migration and invasion of human umbilical vein endothelial cells [77]. Subsequently, Ferris et al. demonstrated that EXOs containing interleukin- 10 (IL-10) and IL-6 secreted by HNSCC and IL-6-dependent inflammatory stimulation resulted in increased angiogenesis [78, 79]. EVs derived from HPV (+) and HPV (-) HNSCC cell lines mainly carried urokinase plasminogen activator (uPA). The urokinase plasminogen activator/urokinase receptor (uPA/uPAR) system is an important pathway that activates pericellular proteolysis, increases vascular permeability, and stimulates angiogenesis by supporting endothelial cells proliferation and migration[80, 81]. Recent studies have shown that EXOs rich in PFKFB3, MMP-13, intercellular cell adhesion molecule-1 (ICAM-1) or thrombospondin-1 (TSP-1) can enhance the release of vascular endothelial growth factor (VEGF-A, IL-8) and then downregulate junction-related proteins (such as claudins), which promote tumour angiogenesis and vascular permeability and become a potential channel system for distant metastasis of tumour cells [42, 50, 77].

Noncoding RNA also plays an important role in tumour angiogenesis; specifically, the level of miRNA-494 is upregulated in OSCC [82]. miRNA-494 is delivered to endothelial cells via EVs secreted by tumour cells. The angiogenic capacity of miRNA-494 is mediated by the phosphatase and tensin homologue (PTEN)-protein kinase B (KAT)-endothelial nitric oxide synthase (NOS) axis. Activated NOS can increase endothelial cells migration and thus promote angiogenesis [83–85]. lncRNA colon cancer-associated transcript 2 (CCAT2) expression is significantly increased in OSCC [86], and it is secreted into EXOs by tumour cells. Subsequently, lncRNA CCAT2 promotes angiogenesis and bcl-2 expression, inhibits bax and caspase-3 expression, and ultimately reduces apoptosis by enhancing VEGFA and TGF-β expression [87]. Therefore, we suggest that EXOs and linc-CCAT2 are putative therapeutic targets in glioma. Functional experiments demonstrated that ephrin-B2 (EPHB2) expression in HNSCC-derived EVs can stimulate EPHB2 reverse signalling, inducing signal transducer and activator of transcription 3 (STAT3) phosphorylation, further regulating angiogenesis [88]. Notably, TDE mediate the delivery of miRNA-9 to inhibit angiogenesis by targeting midkine gene (MDK) and regulating the PDK/AKT pathway NPC. Furthermore, exosomal miRNA-9 levels were positively associated with overall survival, while MDK overexpression was positively correlated with poor prognosis in NPC patients. Thus, we can conclude that miRNA-9 can inhibit tumour angiogenesis, providing a new direction for anticancer treatment [89].

### Roles of microenvironment‐derived EVs in HNSCC

EVs from the tumour microenvironment play a vital role in the development of HNSCC. Cancer-associated fibroblasts (CAFs) are the main stromal cells in the tumour microenvironment (TME) and are indispensable in cancer progression [90]. Mesenchymal stem cells (MSCs), cells undergoing EMT, and tissue-resident cells are the three main cell types that constitute CAFs. miRNA-34a is one of the most important tumour-inhibiting miRNAs. Therefore, the molecular mechanism of its function has been extensively studied [91]. The overexpression of miRNA-34a-5p induced by exosomal metastasis can promote the progression of oral squamous cell carcinoma through the AKT/GSK-3β/β-catenin signaling pathway, thereby inducing epithelial-mesenchymal transition and promoting cancer cell metastasis [92]. Exosomal miRNA-34c is a member of the miRNA-34a family with similar functions and constructs. In vitro and in vivo experiments indicated that overexpression of miRNA-34c inhibit malignant behaviours such as invasion, migration, proliferation and EMT in NPCs by targeting β-Catenin, and in addition, we found alleviated radio resistance upon miRNA-34c overexpression or β-catenin knockdown in NPCs. EXOs derived from MSCs transfected with miRNA-34c showed the same effect. Therefore, exogenous transduction of miRNA-34c to NPC through MSC EXOs can inhibit tumour progression and improve the efficiency of radiotherapy [93]. miRNA-34c also regulates EMT in various tumour cells by directly binding to the mRNAs of SRY-related HMG-box gene (SOX9), special AT rich sequence binding protein (2SATB2), mitogen-activated protein kinase 2 (MAP3K2) [94–98]. Moreover, Peng et al. confirmed that miRNA-34c suppressed exosome shedding by directly targeting RAB27B, thus ending the vicious cycle of intercellular communication mediated by oncogenic EXOs [99]. More and more evidences show that miRNA-34c is a tumour suppressor suppressing miRNA, and it is not limited to HNSCC. CAF-derived EXOs showed excessive transportation of miRNA-382-5p and miRNA-196a to OSCC cells compared with normal fibroblasts and are mainly responsible for the migration and invasion of OSCC cells [60, 100]. In contrast, MSCs-EVs-miRNA-185 significantly reduced cell proliferation and angiogenesis in OSCC tissue and induced apoptosis, indicating their potential role as a novel therapeutic option for OSCC [101, 102]. microfibrillar associated protein 5 (MFAP5)-enriched CAFs promoted oral tongue squamous cell carcinoma (OTSCC) cell growth and migration via activation of the MAPK and AKT pathways mediated by EVs [103]. Tumour cells can be metabolically reprogrammed to adapt to hypoxic circumstances by releasing EVs [104]. Hypoxia induces tumour cells or CAFs to secrete caveolin-1, through trafficking by extracellular vesicles, and contributes to tumour development [40, 75].

#### The effects of tumour‐derived EVs on immune cells

HNSCC is one of the most immunosuppressive human tumours. The role of EVs in HNSCC and immunity has been described extensively in the past decade. In different malignancies, including HNSCC, tumour cells release EVs containing immunoregulatory factors, affecting the TME by mediating immune escape and playing a crucial role in the formation of the premetastatic niche [105, 106]. In this section, we will focus on the vital role of five immune cells (macrophages, dendritic cells, myeloid-derived suppressor cells, natural killer cells, and T lymphocytes) associated with TDE in HNSCC progression and immune escape.

Macrophages, which are derived from monocytes, are considered one of the most important immune cells mainly due to their innate and acquired immune responses to pathogens and prominent positive role in tissue homeostasis. Macrophages show strong plasticity and pluripotency [107, 108]and display significant functional differences under the influence of different microenvironments in vivo and in vitro. Macrophages can be divided into M1-type and M2-type according to their activation state and function [109, 110]. Macrophage uptake of HNSCC-derived EXOs leads to strong regulation of nuclear factor kappa-B (NF-κB), which promotes proliferation, migration, and invasion of tumour cells [111]. Similarly, EXOs induce IL-10 expression in macrophages, thereby inhibiting the development of the immune environment [112]. Hsieh et al. suggested that miRNA-21-abundant TDE was engulfed by CD14 human monocytes, increasing the expression of M2 markers, and inhibiting that of M1 markers. Further investigations revealed that knockout of miRNA-21 in Snail-expressing HNSCC attenuated snail-induced M2 polarization and inhibited angiogenesis and tumour growth [113]. Xiao et al. revealed that macrophages were activated by ingesting EXOs released from OSCC cells via the p38, AKT, and SAPK/JNK signalling pathways. Further evidence showed that thrombospondin 1 (THBS1) derived from OSCC EXOs is involved in the polarization of macrophages towards an M1-like phenotype and creates conditions that promote OSCC progression [114]. MiRNA-9-rich EXOs derived from HPV (+) HNSCC lead to polarization of macrophage M1 via downregulation of peroxisome proliferator-activated receptor δ (PPARδ) and increase the radiosensitivity of tumours [115]. Hence, miRNA-9 may be used as a potential treatment for HNSCC.

Natural killer cells (NK cells) are one of the main cells in the innate immune system. They are not only related to antiviral infection and immune regulation but also play a vital role in antitumour activity [116]. HNSCC-derived EXOs can activate the NF-κB signalling pathway in NK cells to upregulate nucleosome assembly protein 1 (NAP1) expression, promote the expression and phosphorylation of interferon regulatory factor 3 (IRF-3) and release a variety of antitumour inflammatory factors, such as Interferon (IFN), CD40/80/86 [117]. Under certain conditions, EVs also mediate tumour immune escape. HNSCC-derived EVs carrying natural killer receptor G2 (NKG2D) ligands contribute to evading immunity by deceptively weakening the cytotoxicity of NKG2D-mediated NK cells [118–121].

Myeloid-derived suppressor cells (MDSCs) represent a heterogeneous population of immature myeloid cells with immunosuppressive activity [122, 123]. Under hypoxic conditions, HNSCC-derived EVs enhanced the inhibition of MDSCs and attenuated γδT cell activity in a miRNA-21/PTEN/PD-L1 (programmed cell death protein-1) axis-dependent manner, finally inducing the immunosuppressive activity of MDSCs [124]. This finding provides information on the immune checkpoint inhibitor treatment of HNSCC patients.

Dendritic cells (DCs), as powerful antigen-presenting cells, are crucial for the regulation of specific T cell responses in innate antitumour immunity [125]. HPV (+) HNSCC EXOs stimulated DC maturation. In contrast, HPV (-) HNSCC EXOs suppressed DC maturation and the expression of components of the antigen processing machinery [47]. This phenomenon may be associated with a better prognosis for HPV (+) HNSCC. A study by Zhao et al. demonstrated that EXOs derived from 5-aminolevulinic acid photodynamic therapy-treated squamous carcinoma cells can promote DC maturation, which leads to the improvement of antitumour immunity [126]. Moreover, in vitro experiments showed that miRNA-155 loaded in TDE may be a candidate gene for dendritic cell maturation [127, 128].

T lymphocytes are derived from bone marrow pluripotent stem cells. According to different functions in the immune response, T cells can be divided into several subgroups, such as helper T cells (Ths or CD4 + cells), regulatory T cells (Tregs), effector T cells (Tes), cytotoxic T cells (Tcs or CD8 + cells) and memory T cells (Tms) [129, 130]. Plasma-derived EXOs from HNSCC patients contained TGF-β, OX40 (CD134), OX40L(CD134L), and HSP70. These biomolecules induced apoptosis and suppressed CD8 + T cell activation and proliferation by participating in the extrinsic and intrinsic apoptosis pathways, thereby regulating the immune response and driving the tumourigenic process [47, 131]. HNSCC often induce profound immunosuppression, which contributes to disease progression and interferes with immune-based therapies. The presence, quantity, and molecular content of isolated, plasma-derived EXOs can discriminated patients with HNC with active disease (AD) from those with no evident disease (NED) after oncologic therapies [132]. The EXOs of AD patients may contain more inhibitory compounds TGF-β, PD-1 and cytotoxic T lymphocyte antigen 4 (CTLA-4) and cyclooxygenase 2 (COX2) [133–135]. EXOs of patients with AD were significantly more effective than EXOs of patients with NED in inducing apoptosis of CD8 T cells, suppression of CD4 T-cell proliferation, and upregulation of regulatory T-cell (Treg) suppressor functions [132, 136]. The immunosuppression induced by EXOs is related to the disease activity of HNC, suggesting that plasma EXOs can be used as biomarkers of HNSCC progression. EVs derived from HNSCC can not only inhibit the activation and proliferation of T lymphocytes but also prevent their differentiation and promote their conversion to Tregs and MDSCs [137, 138]. Compared with other T-cell classes, Tregs are more susceptible to mediation by tumour-derived EVs, resulting in increased production of immunosuppressive adenosine [43]. HNSCC-derived exosome-enriched galectin-1 decreases the expression of CD27/28-induced CD8 + T cells, displaying a suppressor phenotype [139]. Similarly, hypoxia can lead to the overexpression of exosomal miRNA-24-3p in HNSCC, which can participate in tumorigenesis by inhibiting fibroblast growth factor 11 (FGF 11)-mediated T cell suppression, and may serve as a potential prognostic biomarker for nasopharyngeal carcinoma [140]. Muller et al. demonstrated that EXOs derived from HNSCC cells or plasma of patients control T cell function through surface contact by inducing Ca^2+^ influx into recipient T cells [52]. In addition, NPC-EVs increased the expansion of Tregs, inducing the generation of Tim3 (low) Tregs with increased expression of CD25 and forkhead/winged helix transcription factor (FOXP3) [141]. In conclusion, these findings provide a novel antitumour immune response and strategies for immune cell dysfunction in HNSCC therapy.

#### Role of EVs in HPV-associated HNSCC

The incidence of HPV (+) HNSCC has risen sharply in recent decades, while HPV (-) HNSCC continues to decline [142, 143]. Fortunately, HPV (+) HNSCC responded better to treatment and had a significantly better prognosis than HPV (-) HNSCC [144]. The reasons for this difference are closely related to the communication between EVs and cells in the TME. The HPV (+) cell lines not only produced EXOs carrying the E6/E7 protein but also produced EXOs carrying retinoblastoma (Rb) and survivin protein, whereas the EXOs released by HPV (-) cells did not. Subsequently, these researchers also isolated EXOs through miniSEC from the plasma of HPV (+) and HPV (-) HNSCC patients, and the results showed that plasma contained similarly high levels of exosomal proteins and similarly induced apoptosis of CD8 (+) Jurkat cells or inhibited the proliferation of CD4 (+) T cells; However, only EXOs from HPV (+) tumours had T-cell stimulation [47]. As mentioned above, HPV (+) HNSCC EXOs stimulated DC maturation. In contrast, HPV (-) HNSCC EXOs suppressed DC maturation, which is critical for the good prognosis of HPV (+) HNSCC. Several studies also confirmed this conclusion [47, 145, 146]. In another study, we found that miRNA-27a-3P and miRNA-27b-3P were enriched in EVs produced by HPV (+) and HPV (-) cells. The most abundant miRNA in HPV (+) EVs was miRNA-363-3p [147]. Notably, in OSCC cells expressing miRNA-363-5p, cell proliferation decreased by 40–50 % [148]. These results suggest that intercellular communication mediated by HPV (+) EVs might play a dominant role in antitumour immune responses and inhibit tumour proliferation, which may provide a new treatment for HPV (+) head and neck squamous cell carcinoma.

### The role of EVs in the resistance of HNSCC to radiotherapy and chemotherapy

One of the most destructive issues in HNSCC treatment is the rapid development of drug resistance. In recent years, EVs have become the dominant method of intercellular communication [149]. Cisplatin-based chemotherapy regimens are the first-line treatment for HNSCC therapy and are mostly used in combination with 5-Fluorouracil (5-FU) or taxane [150]. Increased EVs production has been observed in both de novo (H314) and adaptive (H103/cisD2) resistant strains compared with sensitive H103 cells. Protein profiles of these EVs showed that both H103/cisD2- and H314-resistant H103/cisD2 cells downregulated the metal ion transporter ATP1B3 in EVs, indicating a change in drug delivery [151]. This finding suggests that control of EV secretion could be a potential strategy to enhance the efficacy of cancer treatment. Moreover, TDE rich in miRNA-196a and miRNA-21 mediated cisplatin resistance of oral squamous cell carcinoma by targeting PTEN/PDCD4 (programmed cell death 4) and (cyclin dependent kinase inhibitor 1B)CDKN1B/ING5(inhibitor of growth5), respectively [71, 152, 153]. The molecular targeted drug cetuximab is a monoclonal antibody against EGFR IgG1, which can effectively treat locally advanced or recurrent/metastatic HNSCC [154]. EGF stimulation of OSCC cells increased the secretion of EGFR-EVs and EMT. Cetuximab has a 5-fold higher affinity for EGFR than EGF and thus can block the interaction between EGF and EGFR, inhibit the downstream RAS-MEK-ERK signalling pathway and weaken EGF-driven EMT progression, although not completely. Coincidentally, cetuximab was secreted with EGFR-EVs by OSCC cells, identifying a mechanism underlying incomplete inhibition of EMT and cetuximab resistance [74, 155, 156]. Erlotinib is another oral tyrosine kinase inhibitor of the EGFR pathway. Among miRNANAs in EVs derived from erlotinib-resistant cells, miRNA-7704, miRNA-21-5p and miRNA-3960 were significantly upregulated. Furthermore, by performing qRT-PCR and Western blot analysis, vimentin was found to play a key role in regulating erlotinib resistance [157].

Radiotherapy is a typical and aggressive method to treat many locally advanced tumors, but the inherent and acquired drug resistance of cancer cells is the main obstacle to radiotherapy [158]. More and more evidence shows that radiation can induce changes in the miRNA and gene profiles of many tumours [159]. However, the underlying molecular mechanism of radiation resistance is still not fully understood. A non-negligible reason is the extracellular vesicle-mediated radio resistance secreted by HNSCC. Angiogenesis is an important factor leading to radio resistance of malignant tumours [160, 161]. The study of Zheng et al. confirmed that miRNA-23a in extracellular vesicles is significantly up-regulated and mediates the down-regulation of PTEN when malignant tumour cells are exposed to X-ray irradiation, and that the down-regulation of PTEN plays an important role in the enhancement of pro-angiogenesis [162]. This finding implies that the miRNA-23a/PTEN axis is a new therapeutic target for radiotherapy of malignant tumours. The PI3-K/AKT pathway is a carcinogenic pathway with frequent mutations in HNSCC and is a key regulator of radiation resistance and a key driver of cell motility and migration [163, 164]. EXOs derived from irradiated HNSCC cells trigger the PI3-K/AKT pathway to promote migration and increase chemotaxis of recipient cancer cells [165]. Activation of this pathway can be caused by stimulation of receptor tyrosine kinases, such as epidermal growth factor receptor (EGFR). Therefore, we can look for molecules sensitive to EGFR direct treatment to indirectly inhibit the activation of this pathway and improve the efficacy of radiotherapy. The TGF-β superfamily participates in epithelial-mesenchymal transition and maintains the stem cell part, which in turn plays an important role in resistance to radiation and chemoresistance [166, 167]. The study by Dorival et al. emphasized that the level of TGF-β3 protein in extracellular vesicles released from HNSCC cells is a strong predictor of response to chemoradiation therapy [168]. circRNA has tissue and disease specificity, so it can be a potential disease diagnostic biomarker. In the RT-qPCR analysis of circRNA of malignant tumour cells, we found that The expression level of circATP8B4 was significantly higher in RR‑EVs (EVs from radioresistant U251 cells) than this level in Nor‑EVs (EVs from U251 cells). Thus, circATP8B4 from EVs could be a potential biomarker for glioma radio resistance. An in vitro experiment used continuous centrifugation to separate EXOs from conditioned media of irradiated and unirradiated head and neck cancer cells. Quantitative analysis using NanoSight technology indicated an increased exosome release and its role in promoting survival is more obvious from irradiated compared to non-irradiated cells 24 hours after treatment [169]. These findings reveal that EVs are a useful research objects for better understanding radiotherapy resistance in head and neck tumours.

#### Application of EVs in therapeutic strategies

As a natural intercellular information carrier, EVs have broad application prospects in the field of tumour treatment due to their low immunogenicity, loading and modification abilities and good biocompatibility [170]. At present, the application of EVs in clinical therapy is mainly divided into the following several aspects. HNSCC-derived EVs can not only release a variety of antitumour inflammatory factors (IFN, CD40/80/86 and CXCL) but also upregulate the expression of NAP1 and IRF-3 in NK cells, which play an important role in mediating antitumour immunity [117]. Type X collagen gene (COL10A1) has been found to have increased expression in various tumour types [171]. Xie et al. demonstrated that COL10A1 was upregulated; however, miRNA-101-3p was downregulated in HNSCC tissues and cell lines, and a dual-luciferase reporter gene assay confirmed that miRNA-101-3P targets COL10A1. Subsequently, EXOs derived from human bone marrow mesenchymal stem cells (hBMSCs) were isolated and cocultured with tumour cells, and the results showed that EXOs derived from hBMSCs overexpressing miRNA-101-3p could inhibit the progression of tumours [101]. In addition, in vitro experiments further confirmed the inhibitory effect of hBMSC-derived EXOs carrying miRNA-101-3p on tumour cell invasion and migration [101]. miRNA-138 has been shown to target CTLA-4 and PD-1 in CD4 + T cells, thus playing an anticancer role [172]. Following the demonstration that γδ T cell-derived extracellular vesicles (γδTDEs) as the drug delivery system for miRNA138 can hinder the development of HNSCC, Li et al. found that miRNA-138 delivered by TDE had a synergistic inhibitory effect on (Centre Antoine Lacassagne-27) CAL-27 cells in nude mice. Tumour growth in OSCC (SCCVII) cells was inhibited in C3H mice treated with miRNA-138 TDE but not in T-deficient nude mice [173]. γδTDEs with miRNA-138 increased IFN-gamma production, CD8 + T cell proliferation, and cytotoxicity against OSCC cells [173]. Curcumin is the bioactive ingredient of turmeric and is known for its anticancer effects [174]. Recently, several studies have shown that curcumin, doxorubicin and paclitaxel can be passively loaded into EVs to improve their therapeutic efficacy [175–177]. The researchers loaded curcumin into Candida galbrata EVs and then transferred it into the OTSCC cell line and compared it with the unloaded curcumin cell line. The addition of curcumin improved bioavailability, and the anticancer effect on OTSCC cells unfortunately did not increase [178]. However, paclitaxel-loaded EVs have been shown to have antitumorigenic effects [179, 180], with doxorubicin and paclitaxel-loaded EVs demonstrating the ability to cross the blood-brain barrier in zebrafish. This finding shows that EXOs derived from brain endothelial cells can be used as a carrier for intracerebral administration to treat brain cancer [177]. Another application of EVs for therapeutic intervention is tumour vaccination. As an important intercellular communication tool and distinct biomarker associated with these vesicles, TDE can also be applied in vaccine immunotherapy [181]. Antigen-presenting EXOs from B lymphocytes and DCs containing major histocompatibility complex I/II (MHCI/II) complexes could stimulate CD4 + and CD8 + T cells as therapeutic HPV vaccines [182]. Kanuma et al. also demonstrated that CD63-mediated antigen delivery into EVs via DNA vaccination leads to strong CD8 T cell responses [183]. Therefore, the experimental validation studies described above indicate that EXOs hold promise as nano delivery vehicles for cancer treatment.

#### EVs and biomarkers

**Fig. 3:**
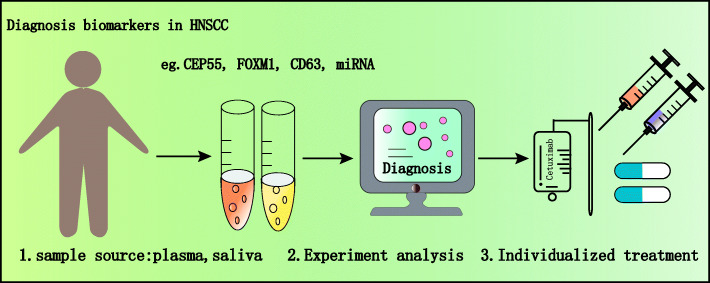
Exosomes derived from blood or saliva have been tested in the laboratory. Exosomes carrying or highly expressing miR-10b-5p, miR-486, miR-517b-3p, miR-302b-3p, CEP55, FOXM1 and CD63 can be used HNSCC potential biomarkers for diagnosis, personalized treatment, and prognosis evaluations

One of the most exciting applications of EVs research in cancer is their potential use as biomarkers because they are in body fluids. EVs-based diagnostics are suggested to be optimal candidates for non-invasive diagnosis [184] (Fig. 3). Qadir et al. demonstrated that exosomal Centrosomal protein 55 (CEP55) and forkhead box protein M1 (FOXM1) mRNA cargos in blood might be exploited as a cancer biomarker for a non-invasive mode of diagnosis and prognosis of HNSCC [185]. For 10 OSCC plasmatic EXOs, surgical treatment induced a dramatic reduction of the plasmatic levels of EXOs expressing CD63. Subsequently, statistical analysis demonstrated that lower levels of plasmatic EXOs were correlated with a better life expectancy of OSCC patients [186]. This finding suggested that expression level analysis of CD63 using plasmatic-secreted EXOs could be beneficial for predicting the prognosis of HNSCC in clinical settings. Ogawa et al. first discovered EXOs in saliva in 2008. Salivary EXOs have the advantage of being simple and non-invasive to collect compared with plasma EXOs; they also contain less protein than blood, so their identification and quantification can be substantially simplified [187, 188]. In terms of storage, they can be preserved at 4 °C or -80 °C, making it easier to use them in a clinical setting [189]. Langevin et al. comprehensively identified miRNA sequences of EXOs from 4 HNSCC cell lines and oral epithelial control cells using a new generation miRNA sequencing technique, and their studies showed that many miRNA were shared in salivary EXOs from healthy and cancerous cells. However, compared with those in the control group, the exosomal miRNA-486-5p, miRNA-486-3p and miRNA-10b-5p from HNSCC cell lines were strikingly higher [190]. Similar studies reported that miRNANA-21, miRNANA-184, miRNA-412-3p, miRNA-512-3p, miRNA-27a-3p, and miRNA-494-3p in salivary EXOs of OSCC patients were significantly higher than those of the healthy control group. In addition, miRNA-302b-3p and miRNA-517b-3p were only expressed in salivary EXOs of OSCC patients [191–193]. High expression of CD63 was also found in salivary EXOs from patients with HNSCC [194, 195]; moreover, exosomal PPIA + was downregulated as a poor prognostic factor in the saliva of OSCC patients [196]. All these results show that saliva and plasma-derived EXOs could be used as potential diagnostic, treatment, and prognostic biomarkers. Moreover, a growing number of experiments have confirmed that salivary components can also be used to monitor and screen other tumours, such as pancreatic cancer [197], lung cancer [198], and breast cancer [199].

## Conclusions

In summary, EV research is an attractive emerging field, whose advantages depend on their accessibility and specific DNA/RNA/protein profiles as well as their important role in tumour progression. Identifying these genomic profiles can be used to assess various biomarkers for the early detection of HNSCC. The extracellular vesicles in HNSCC can not only assess the degree of malignancy and tumour progression but also provide appropriate methods and monitors for the treatment of HNSCC. However, EVs in HNSCC can also assess the degree of malignancy and progression of the tumour. In addition, EV-mediated cancer vaccines have recently made some progress. EVs act as a “double-edged sword” and are closely related to the malignant invasion and tumour resistance of HNSCC. On the one hand, EVs can prepare the soil for tumour seeding and create a suitable TME. On the other hand, EVs, as “leagues” of tumours, can not only promote the proliferation and metastasis of cancer cells but also facilitate the immune escape or drug resistance of cancer cells. Although these studies have prompted the clinical applications of EVs, many problems need to be further elucidated. First, there is a debate about the definition of EVs and classification of subtypes and lack of standardization and unification of extraction techniques, which is a tricky issue for studies of EVs. Second, there are multiple bioactivators in EVs, and what are the main functional components in EVs? Third, although RNAs have been the focus of EVs in HNSCC for the last decade, which component may be the most suitable for biomarker identification? Further research is needed to elucidate the basic mechanisms/characteristics of EVs biology in HNSCC. Due to the key role of EVs in carcinogenesis, more research is needed in this field to explore the potential of EXOs in tumour treatment.

## Data Availability

Not applicable. Not applicable. Yes.

## Abbreviations

EVs: Extracellular vesicles
HNSCC: Head and neck squamous cell carcinoma
EXO: Exosomes
MVs: Microvesicles
ESCRT: Endosomal sorting complex required for transport
MVBs: Multivesicular bodies
GFP: Green fluorescent protein
miRNAs: microRNAs
LncRNAs: Long noncoding RNAs
HPV: Human papillomavirus
TRAP1: TNF receptor associated protein
TNF: Tumour necrosis factor
NPC: Nasopharyngeal carcinoma
EGFR: Epidermal growth factor receptor
EGF: Epidermal growth factor
HSP-90: Heat shock protein 90
Dsg-2: Desmonglein-2
MMP: Matrix metalloprotein
HOTAIR: HOX transcript antisense RNA
BCL2: B-cell lymphoma 2
PGE2: Prostaglandin E2
TGF-β: Transforming growth factor-β
PFKFB3: 6-phosphofructo-2-kinase/fructose-2,6-biphosphatase
CD63: Cluster of differentiation 63
Shh: Sonic Hh
IL-10: Interleukin-10
TSGA10: Targeting testis-specific gene antigen
AKT: Protein kinase B
MAPK: Mitogen-activated protein kinase
NF-κB: Nuclear factor kappa-B
ERK: Extracellular signal-regulated kinase
JNK: c-Jun N-terminal kinases
DENND2D: DENN/MADD Domain Containing 2D
EBV: Epstein-Barr virus
UCH-L1: Ubiquitin C-terminal hydrolase L1
LOXL2: Lysyl oxidase like 2
OSCC: Oral squamous cell carcinoma
uPA: Urokinase plasminogen activator
uPA/uPAR: Urokinase plasminogen activator/urokinase receptor
ICAM-1: Intercellular cell adhesion molecule-1
TSP-1: Thrombospondin-1
EPHB2: Ephrin-B2
MDK: Midkine gene
EMT: Epithelial-mesenchymal transition
NOS: Nitric oxide synthase
CCAT2: Colon cancer-associated transcript 2
HIF-1α: Hypoxia-inducible factor-1α
COL10A1: Type X collagen gene
CAFs: Cancer-associated fibroblasts
TME: Tumour microenvironment
MSCs: Mesenchymal stem cells
OTSCC: Oral tongue squamous cell carcinoma
NK cells: Natural killer cells
MDSCs: Myeloid-derived suppressor cells
DCs: Dendritic cells
Ths or CD4+cell: Helper T cells
Tregs: Regulatory T cells
Tes: Effector T cells
Tcs or CD8+cell: Cytotoxic T cells
Tms: Memory T cells
CTLA-4: Cytotoxic T lymphocyte antigen 4
PD-1: Programmed cell death
hBMSCs: Human bone marrow mesenchymal stem cells
γδTDEs: γδ T cell-derived extracellular vesicles
TDE: Tumour-derived EVs
